# Surgery

**Published:** 1877-08

**Authors:** 


### II. Surgery.

Nerve-Stretching in Sciatica.—John Chiene (*Practitioner*, May 1877).—The writer reports two cases of sciatica cured by operation as recommended by Prof. Nussbaum, of Munich. The operation consists in exposing, by an incision, the large nerve affected as the sciatic, and lifting it upon the finger. It is then pulled proximally and distally, and finally the limb of the patient is lifted up from the table by the nerve. Both patients were strong, muscular men, employed as furnace men, and, therefore, exposed to great alterations of temperature. One was left-handed, and the other right-handed. The former had sciatica in the right leg, while the latter had it in the left. This can be accounted for by the fact, that supposing the man to be right-handed, the nerve of the left leg is put on the stretch at each time of heaving coals with a shovel into the furnace. The opposite limb is affected if the man is left-handed. This stretching of the nerve as it passes out of the sciatic notch, the writer thinks is probably one of the causes of sciatica.

Both men had suffered severely with pain, and been unable to work for several months. The various remedies, as quinine, injections of morphine, blisters, galvanism, etc., had been tried without any good effect. In each case the sciatic nerve was exposed by an incision over it below its exit from under the fibres of the gluteus maximus. The nerve was then hooked upon the finger and forcibly pulled from above downwards, and from below upwards, and the limb lifted from the table by it. The wound was treated antiseptically, and allowed to heal. The relief of pain was immediate after the operation, motion and sensation of the limb were not affected. Both men were able, the next day after the operation, to move their limbs without pain. At the end of two weeks, they were able to get out of bed and walk about, and in a month were discharged from the hospital cured. The *rationale* of this treatment the writer finds difficult to explain. If neuritis is one of the causes of sciatica, and the pathological conditions of the nerve are adhesions and thickening of the neurilemma, which press upon and irritate the nerve fibrils, then probably the result of severe stretching is to break down the adhesions, and remove the pressure from the nerve. It is worthy of mention, that the relief of pain was immediate after the operation, and the functions of motion and sensation were not disturbed. It may then be inferred, that the nerve tissue proper was not ruptured, but the stretching acted on the neurilemma or insulating part of the nerve.

